# The Impact of Pet Ownership on Older Single Expatriate Females in Panamá

**DOI:** 10.1093/geroni/igaf122.3080

**Published:** 2025-12-31

**Authors:** Melissa Mansfield

**Affiliations:** The Gerontechnology Group, Houston, Texas, United States

## Abstract

This qualitative hermeneutic phenomenological study explored the lived experience of singlehood and pet companionship among expatriate women aged 55 and older in the Panamá Oeste Province. The research aimed to understand how pet companionship shapes the aging experience and impacts mental health, relationships, and loneliness for this demographic. Semistructured interviews were conducted with 15 single expatriate women who were current or prior pet owners, supplemented by participant observation and photo elicitation techniques. Four themes emerged from the interpretative phenomenological analysis: (a) companionship and emotional bonds, (b) daily structure and routine, (c) personal security and safety, and (d) challenges of pet ownership. Findings suggest pet companionship significantly enhances the aging experience, positively impacting mental health, social connections, daily structure, security, and independence. The study contributes to understanding human–animal bonds in later life, particularly in expatriate contexts. It also supports attachment theory and activity theory of aging as frameworks for understanding how pet companionship influences overall well-being and social engagement in a cross-cultural context. Results highlight the multifaceted role of pets in the lives of older expatriate women living alone in Panamá. Future researchers should investigate the experiences of single expatriate men with close pet relationships across diverse cultural settings to broaden the understanding of this phenomenon.

